# Acute Phase Proteins Are Baseline Predictors of Tuberculosis Treatment Failure

**DOI:** 10.3389/fimmu.2021.731878

**Published:** 2021-11-15

**Authors:** Nathella Pavan Kumar, Kadar Moideen, Arul Nancy, Vijay Viswanathan, Kannan Thiruvengadam, Shanmugam Sivakumar, Syed Hissar, Hardy Kornfeld, Subash Babu

**Affiliations:** ^1^ Indian Council of Medical Research (ICMR)-National Institute for Research in Tuberculosis, Chennai, India; ^2^ National Institute for Research in Tuberculosis, International Center for Excellence in Research, National Institutes of Health, Chennai, India; ^3^ Department of Diabetology, Prof. M. Viswanathan Diabetes Research Center, Chennai, India; ^4^ Department of Medicine, University of Massachusetts Medical School, Worcester, MA, United States; ^5^ Laboratory of Parasitic Diseases, National Institute of Allergy and Infectious Diseases, National Institutes of Health, Bethesda, MD, United States

**Keywords:** tuberculosis, acute phase proteins, biomarker, inflammation, TB treatment

## Abstract

Systemic inflammation is a characteristic feature of pulmonary tuberculosis (PTB). Whether systemic inflammation is associated with treatment failure in PTB is not known. Participants, who were newly diagnosed, sputum smear and culture positive individuals with drug-sensitive PTB, were treated with standard anti-tuberculosis treatment and classified as having treatment failure or microbiological cure. The plasma levels of acute phase proteins were assessed at baseline (pre-treatment). Baseline levels of C-reactive protein (CRP), alpha-2 macroglobulin (a2M), Haptoglobin and serum amyloid P (SAP) were significantly higher in treatment failure compared to cured individuals. ROC curve analysis demonstrated the utility of these individual markers in discriminating treatment failure from cure. Finally, combined ROC analysis revealed high sensitivity and specificity of 3 marker signatures comprising of CRP, a2M and SAP in distinguishing treatment failure from cured individuals with a sensitivity of 100%, specificity of 100% and area under the curve of 1. Therefore, acute phase proteins are very accurate baseline predictors of PTB treatment failure. If validated in larger cohorts, these markers hold promise for a rapid prognostic testing for adverse treatment outcomes in PTB.

## Introduction

Systemic inflammation is a characteristic hallmark of pulmonary tuberculosis (PTB) (1–3). Systemic inflammation is typically characterized by elevations in the levels of acute phase proteins, including C-reactive protein (CRP), apha-2 macroglobulin (a2M), haptoglobin (Hp) and serum amyloid P (SAP) (4–7). Several studies have associated increased circulating levels of these acute phase proteins with PTB and indeed, CRP is often used a point-of-care test to aid in the diagnosis of PTB (8–13). Studies have also reported that haptoglobin is clinically a relevant host biomarker for TB diagnosis and disease progression (14).

While standard TB treatment is associated with high rates of favorable outcomes (recurrence free microbiological cure), a sizeable proportion of patients experience adverse outcomes in the form of treatment failure or recurrence after treatment (15). We have previously reported that plasma chemokine signatures (16), as well as Matrix metalloproteinases (MMPs) and tissue inhibitors of matrix metalloproteinases (TIMPs) (17) can be used as novel biomarkers for predicting adverse treatment outcomes like failure, relapse, and death in individuals with PTB. The host factors that drive this dichotomy in drug – sensitive TB are ill defined (18). We hypothesized that TB treatment failures would be driven by heightened systemic inflammation at baseline. To test this hypothesis, we examined the baseline levels of acute phase proteins in a nested case-control study of TB treatment failure versus cure in a cohort of PTB individuals in Chennai, India. Our results show that acute phase proteins are baseline predictors of treatment failure.

## Materials and Methods

### Ethics Statement

This study was approved by the Ethics Committees of the Prof. M. Viswanathan Diabetes Research Center (ECR/51/INST/TN/2013/MVDRC/01) and NIRT (NIRT-INo:2014004). Informed written consent was obtained from all participants. All the methods were performed in accordance with the relevant institutional ethical committee guidelines.

### Study Population

Participants were enrolled from the Effect of Diabetes on Tuberculosis Severity (EDOTS) study, a prospective cohort study conducted in Chennai, India (19). The inclusion criteria were new smear and culture positive adults between 20 and 75 years of age. The exclusion criteria were previous TB history or treatment, drug resistant TB, more than one week of TB treatment currently, pregnancy or lactation, HIV positivity or on immunosuppression. The diagnosis of pulmonary TB was established by positive sputum culture on solid media with compatible chest x-ray. Anti-TB treatment (ATT) was managed by government clinics in Chennai according National Tuberculosis Elimination Program standards, which is based on the Directly Observed Treatment Short Course (DOTS) therapy. Participants were followed up monthly through the six-month course of treatment. We conducted a nested case-control study with microbiological TB treatment failure matched in a 1:2 ratio to microbiological cure. Cure was defined as negative sputum cultures at months 5 and 6 of treatment. Treatment failure was defined as positive sputum culture at months 5 or 6. There was a total of 18 treatment failures and 36 cured controls. Case – control matching was carried out on the basis of age, gender, body mass index and diabetic status. Peripheral blood was collected in heparinized tubes. Following centrifugation, plasma was collected and stored at -80°C till further analysis. Sample collection was performed at baseline (before treatment initiation) in all participants.

### Acute Phase Proteins

Plasma levels of alpha-2 macroglobulin (A2M), C-reactive protein (CRP), haptoglobin and Serum Amyloid P (SAP) were measured using a Milliplex MAP Human CVD Panel Acute Phase magnetic bead panel 3 from Millipore, using a multiplex platform according to the manufacturer’s instructions. The lowest detection limits for acute phase proteins was as follows: alpha-2 macroglobulin (A2M); 0.49 ng/mL; C-reactive protein (CRP), 0.05 ng/mL; haptoglobin, 0.06 ng/mL; and Serum Amyloid P(SAP) 0.06 ng/mL.

### Statistical Analysis

Geometric means (GM) were used for measurements of central tendency. Differences between the two groups were analyzed using the Mann-Whitney test. Receiver Operator Characteristics (ROC) curves was designed to test the power of each candidate biomarker to distinguish treatment failures from cured individuals. Analyses were performed using Graph-Pad PRISM Version 9.0. P values < 0.05 were considered statistically significant. Computation and selection of optimal biomarker combinations by integrative ROC were analysed using freely available web application (http://CombiROC.eu) CombiROC v.1.2. Classification and regression trees (CART) model were employed to identify the cut-off for the biomarkers which separate the TB treatment failure and cure. The analysis was done using the R (R Foundation for Statistical Computing, Vienna, Austria) software.

## Results

### Study Population

The demographics of the study population are shown in **Table 1**. The median age was 45 (interquartile range [IQR] 38-51) years for treatment failures and 45 (IQR 39-53) years for cured individuals. There were no significant differences in gender, BMI, diabetic status, lipid profile, smoking or alcohol use (**Table 1**). There were also no differences in smear or culture grades or presence of cavities between the two groups (**Table 1**).

**Table 1:** Demographic and clinical characteristics of the study population.

	Cure (n=36)	Treatment failures (n=18)	Sig.
Age (in Years)	45.0 (38.0 – 50.5)	45.0 (39.0 – 53.0)	0.818
Gender			
Female	7 (19.4)	2 (11.1)	0.439
Male	29 (80.6)	16 (88.9)	
BMI	17.0 (15.4 - 19.4)	16.8 (14.9 - 19.6)	0.646
Diabetes			
Non-Diabetes	14 (38.9)	5 (27.8)	0.420
Diabetes	22 (61.1)	13 (72.2)	
Cough Duration	5.0 (3.0 - 8.0)	4.5 (4.0 - 8.0)	0.661
Cough			
Absence	2 (5.6)	0 (0)	0.308
Presence	34 (94.4)	18 (100)	
Dyslipidaemia			
Absence	36 (100)	18 (100)	NA
Presence	0 (0)	0 (0)	
Smoking			
Never	20 (55.6)	6 (33.3)	0.123
Past/Current	16 (44.4)	12 (66.7)	
Alcohol			
Never	13 (36.1)	4 (22.2)	0.300
Past/Current	23 (63.9)	14 (77.8)	
Cavity			
Absence	22 (61.1)	10 (55.6)	0.345
Presence	14 (38.9)	8 (44.4)	
Smear			
1+	25 (69.4)	10 (55.6)	0.561
2+	9 (25)	6 (33.3)	
3+	2 (5.6)	2 (11.1)	
Culture			
1+	11 (30.6)	6 (33.3)	0.185
2+	12 (33.3)	2 (11.1)	
3+	13 (36.1)	10 (55.6)	

Values were presented as n (%) and median (first - third quartile); Fisher Exact and Mann-Whitney test were used to check the significance

### Treatment Failures in PTB Are Characterized by Increased Systemic Inflammation

**Figure 1:**
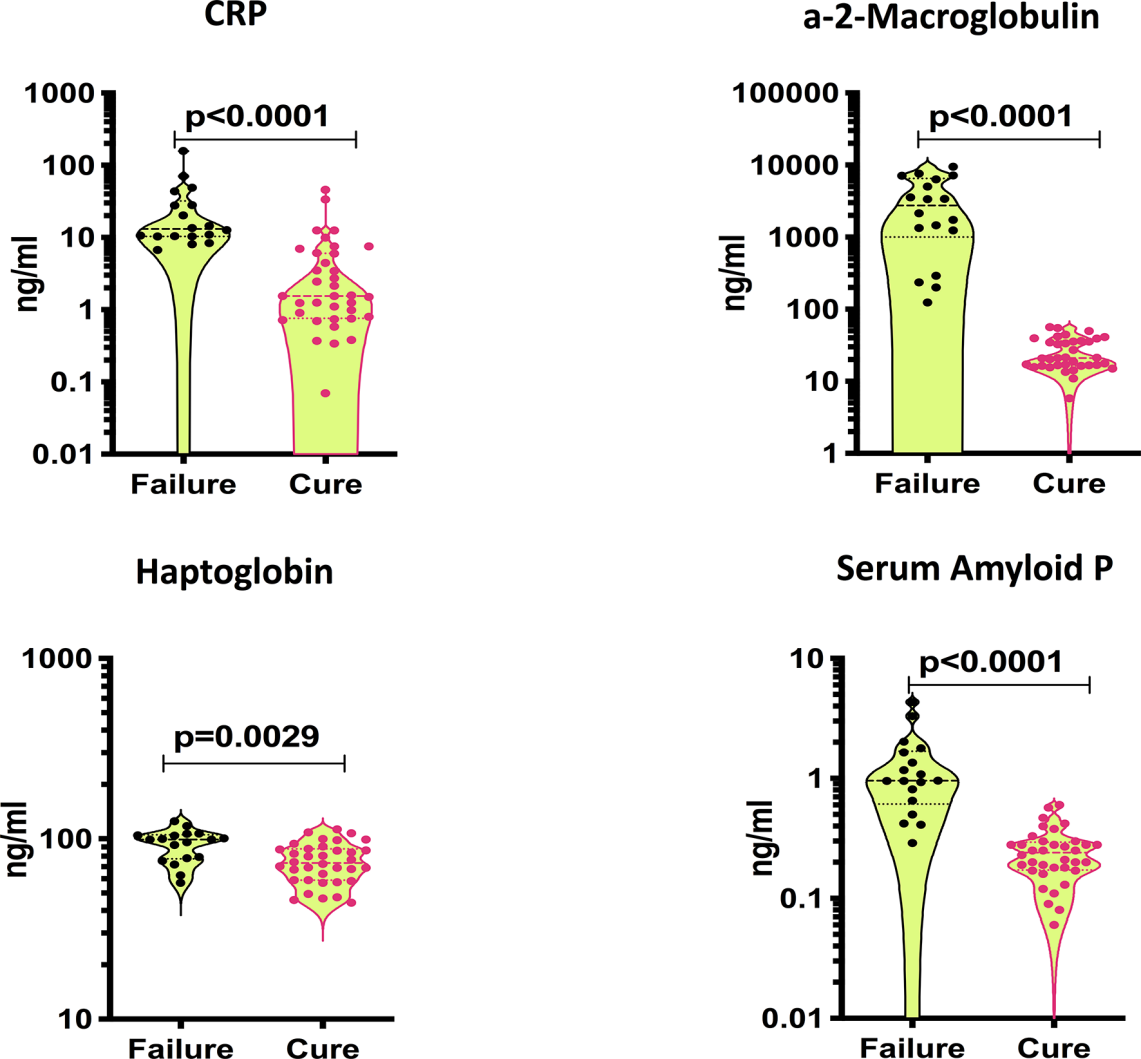
Elevated baseline plasma levels of APPs in TB treatment failure. The baseline plasma levels of APPs were measured in failure (n=18) and cure (n=36). The data are represented as violin plots with each circle representing a single individual. P values were calculated using the Mann-Whitney test with Holm’s correction for multiple comparisons.

To assess baseline systemic inflammation in treatment failure and cured individuals, we measured the levels of acute phase proteins at baseline (pre-treatment). As shown in **Figure 1**, the levels of CRP (Geometric Mean (GM) of 18.34 ng/ml in failure *versus* 2 ng/ml in cure), A2M (GM of 1866 ng/ml in failure *versus* 23.4 ng/ml in cure), Hp (GM of 91.2 ng/ml in failure *versus* 72.9 ng/ml in cure) and SAP (GM of 1.02 ng/ml in failure *versus* 0.22 ng/ml in cure) were significantly higher in cases compared to controls. Thus, adverse treatment outcomes in PTB are associated with increased baseline levels of acute phase proteins.

### Treatment Failures in PTB Are Marked by a Three-Marker Signature of Acute Phase Proteins

**Figure 2:**
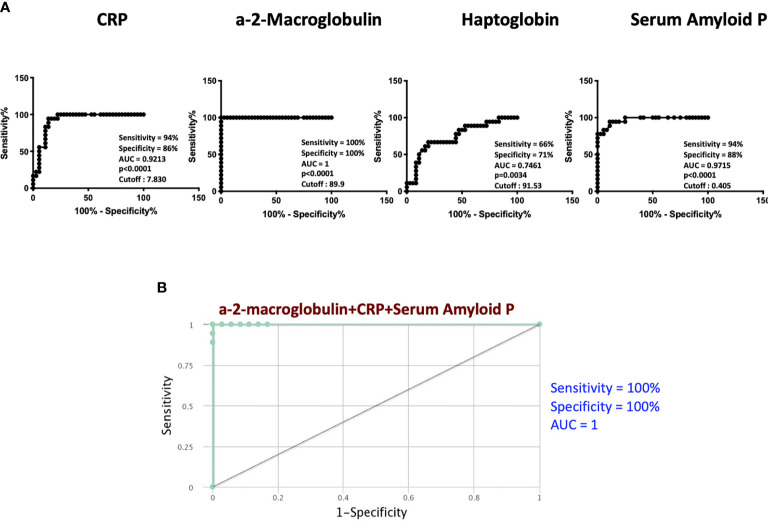
ROC analysis to estimate the discriminatory power of APPs in TB treatment failure. **(A)** ROC analysis to estimate the sensitivity, specificity and AUC was performed using a-2M, CRP, Hp and SAP to estimate the capacity of these markers to distinguish individuals with failure *vs*. Cure. **(B)** Combination of ROC model analysis shows the APPs that exhibited the highest accuracy in discriminating failure and cure.

To determine if we could utilize acute phase proteins as individual biomarkers for treatment failures *versus* cured individuals, we performed individuals ROC analysis on acute phase proteins. As shown in **Figure 2A**, ROC analysis of CRP (Sensitivity 94%, Specificity 86% and AUC=0.9213), Hp (Sensitivity 66%, Specificity 71% and AUC=0.7461) and SAP (Sensitivity 94%, Specificity 88% and AUC=0.9715) showed significantly high AUC with sensitivity and specificity, especially for A2M, which exhibited 100% sensitivity and specificity. To determine if we could derive a signature of acute phase proteins that could be used as a biomarker for treatment failures *versus* cured individuals, we performed combined ROC analysis on 3 acute phase proteins. As shown in **Figure 2B**, combiROC analysis of CRP, A2M and SAP exhibited a high AUC (1) with 100% sensitivity and specificity in differentiating treatment failures from microbiologic cures. Thus, treatment failures are marked by a three-marker signature of acute phase proteins.

### Biomarkers Discriminating Favorable From Unfavorable TB

**Figure 3:**
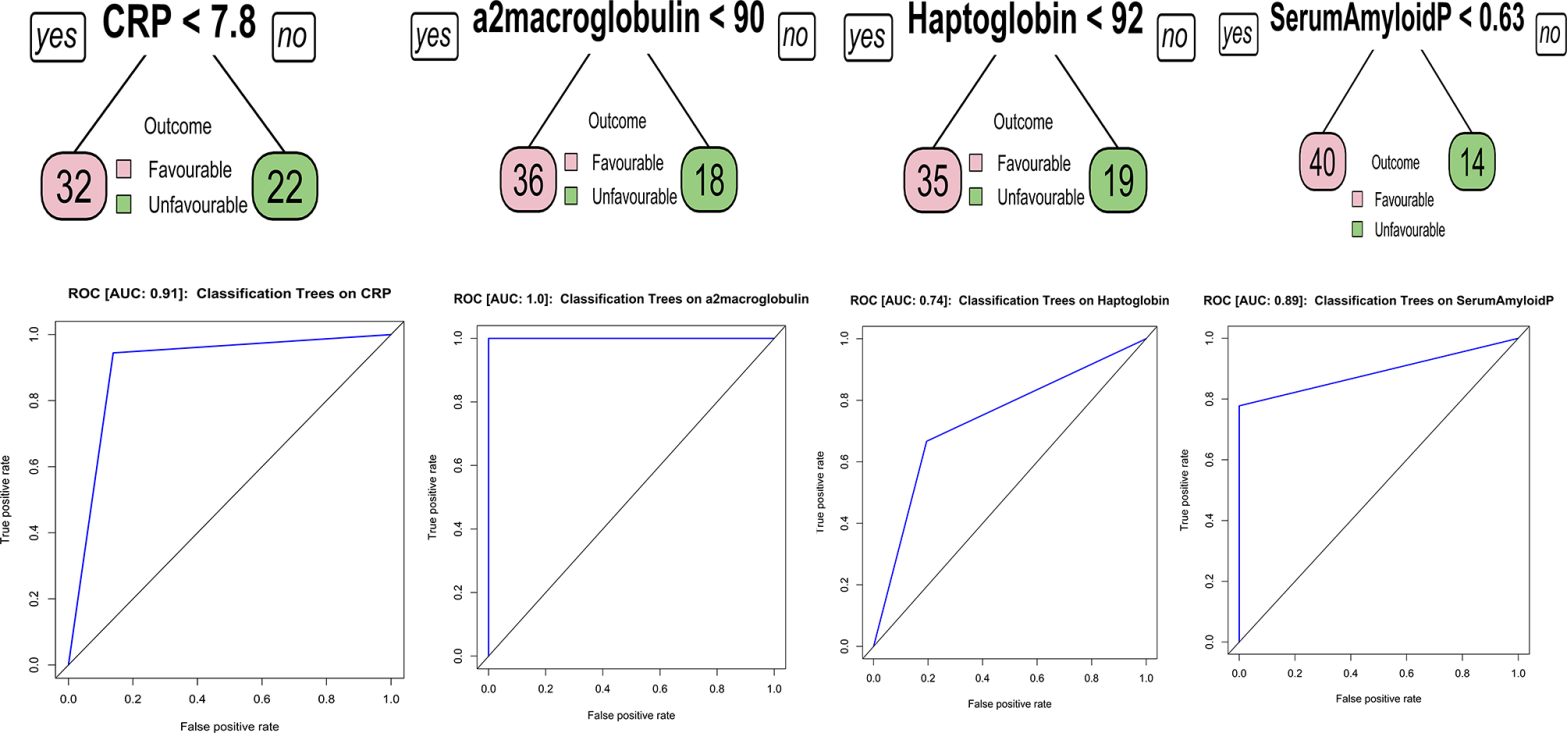
Identification of biomarkers showing the strongest associations with active TB disease. CART model analysis shows the APPs that exhibited the highest accuracy in discriminating TB treatment failure from cure. Receiver operator characteristics curves were employed to quantify the accuracy of single biomarkers.

Classification and regression trees (CART) models were employed to identify the cut-off for the biomarkers which separate the treatment failures from cure. As input for tree construction, we used data on all the markers and selected the most relevant biomarker that classifies the group more accurately (**Figure 3**). Briefly, the data set formed a parent node, which contains the whole population. The best peak to separate the data set was selected. As shown in **Figure 3**, CRP with a cut-off value of 7.8 ng/ml with AUC:0.91, A2M with a cut-off value of 90 ng/ml with AUC:1, Hp with a cut-off value of 92 ng/ml with AUC:0.74 and SAP with a cut-off value of 0.63 ng/ml with AUC:0.89 was able distinguish failure *vs* cure. This CART analysis was able to demonstrate that APPs such as CRP and a2M act as a sensitive diagnostic immune biomarker for prediction of TB treatment outcomes.

## Discussion

Tuberculosis patients are highly heterogeneous when it comes to the extent of disease, immune activation and vulnerability to treatment failure (18, 20). Therefore, tools guiding individual management and treatment are likely to increase cure rates and be more cost-effective, which would also largely facilitate the clinical trial evaluation of new drugs and regimens. Treatment is prolonged, and patients on ATT often experience treatment failure for various reasons (15). Therefore, there is an urgent need to identify host mechanisms that drive treatment failure, recurrence and death, since these factors in addition to bacterial factors, contribute majorly to adverse treatment outcomes (21). Greater understanding of the host processes underlying this phenomenon could provide clues to developing biomarkers of adverse treatment outcomes in TB.

Our study examined the occurrence of systemic inflammation in a well characterized cohort of PTB individuals followed up until completion of treatment and classified as treatment failures or microbiological cures. Our data reveal that systemic inflammation, as measured by the plasma levels of acute phase protein, at baseline are significant predictive biomarkers of treatment failure. Data from various studies have collectively reported that CRP exhibited high sensitivity (93%) and moderate specificity (60%) in distinguishing active TB from other conditions (22). Indeed, CRP has been recommended as a simple, point-of-care to aid in the diagnosis of PTB (8–13). Therefore, our data on CPR being significantly associated with treatment failures is not surprising. A2M, Hp and SAP have also been reported to be useful biomarkers for TB diagnosis (14, 23–26). Our data clearly demonstrate that all the 4 acute phase proteins are present at higher levels in failures compared to cured individuals. Our ROC analysis data confirms the utility of CRP, A2M and SAP as potentially valuable biomarkers for treatment failure. Finally, we derive a three-biomarker signature comprising of CRP, A2M and SAP that provides 100% sensitivity and 100% specificity in distinguishing treatment failures from cured individuals in our cohort. While systemic inflammation in TB is not a new phenomenon, this to our knowledge is one of the first reports on systemic inflammation being an underlying player in adverse treatment outcomes in PTB.

Our study groups were matched for age, gender, body mass index and diabetic status. Other confounders such as smoking, alcohol use, lipid status, smear and culture grade did not exhibit any significant differences. In addition, our data provide cut off values in pg/ml for the biomarkers of CRP, A2M and SAP to be used for classification of individuals at risk of treatment failure. We anticipate validating these cut off values in future cohorts. Therefore, our study provides an important advance in the field TB biomarkers by providing a potential signature of high utility in baseline stratification of high risk individuals for TB treatment. Our study adds to the growing list of biomarkers that can be used to predict adverse treatment outcomes at pre-treatment timepoints. Further confirmation of these markers in diverse geographic and ethnic setting would add value to their utility in this field. In addition, studies also reported that in other respiratory chronic diseases like chronic obstructive pulmonary disease, asthma and pulmonary fibrosis also persistent inflammation is seen within the respiratory tract underlies the pathogenesis of numerous chronic pulmonary diseases (27, 28)

Our study has certain limitations. The sample size was moderate, the study was performed in a single cohort without external validation and cause-effect relationships were not determined. Our study also has not investigated the causes of the inflammatory condition which is due to the tuberculosis, or which may be due to comorbid conditions. Nevertheless, this data is of major importance in designing future studies to elucidate host biomarkers of adverse treatment outcomes, which would be enormously beneficial in eliminations strategies for PTB. Moreover, our study also contributes to the growing understanding of the pathogenesis of host responses that govern treatment outcomes in TB.

## Data Availability Statement

The original contributions presented in the study are included in the article/supplementary material. Further inquiries can be directed to the corresponding author.

## Ethics Statement

The studies involving human participants were reviewed and approved by the NIRT-Ethics committee. The patients/participants provided their written informed consent to participate in this study.

## Author Contributions

Designed the study (SB, NK). Conducted experiments (NK, KM, AN). Acquired data (NK, KT). Analyzed data (NK, KT). Contributed reagents and also revised subsequent drafts of the manuscript (SH, SS, HK, SB). Responsible for the enrolment of participant and also contributed to acquisition and interpretation of clinical data (VV, SH, HK) Wrote the manuscript (SB, NK). All authors read and approved the final manuscript.

## Funding

This project has been funded in whole or in part with Federal funds from the Government of India’s (GOI) Department of Biotechnology (DBT), the Indian Council of Medical Research (ICMR), the United States National Institutes of Health (NIH), National Institute of Allergy and Infectious Diseases (NIAID), Office of AIDS Research (OAR), and distributed in part by CRDF Global [grant USB1-31149-XX-13]. This work is also funded by CRDF Global RePORT India Consortium Supplemental Funding [grant OISE-17-62911-1].

## Author Disclaimer

The contents of this publication are solely the responsibility of the authors and do not represent the official views of the DBT, the ICMR, the NIH, or CRDF Global. This work was also funded in part by the Division of Intramural Research, NIAID, NIH.

## Conflict of Interest

The authors declare that the research was conducted in the absence of any commercial or financial relationships that could be construed as a potential conflict of interest.

## Publisher’s Note

All claims expressed in this article are solely those of the authors and do not necessarily represent those of their affiliated organizations, or those of the publisher, the editors and the reviewers. Any product that may be evaluated in this article, or claim that may be made by its manufacturer, is not guaranteed or endorsed by the publisher.
